# Supplementary material for: Temporal Feasibility Constraints on Wingbeat‐Call Synchrony in Actively Echolocating Bats
Source: Ecol Evol. 2026 May 14;16(5):e73622. doi: 10.1002/ece3.73622 (PMC13173462; doi:10.1002/ece3.73622)
Supplement: Supplementary file 1 — Data S1: ece373622‐sup‐0001‐supinfo.docx. [file ECE3-16-e73622-s001.docx]

# APPENDIX

**A SUPPLEMENTARY TABLES**

**Table A1: Descriptive statistics for synchronous call counts per run.** Summary statistics are computed across

*N* = 200 sampled simulation runs for each condition.

| Condition | Mean | SD | Median | IQR | P05 | P25 | P75 | P95 | MAD |
| --- | --- | --- | --- | --- | --- | --- | --- | --- | --- |
| C1 (*f_w_* fixed, *θ* fixed) | 10.2 | 5.6 | 9 | 7.0 | 4.0 | 6.0 | 13.0 | 21.5 | 3 |
| C2 (*f_w_* dyn, *θ* fixed) | 24.9 | 12.3 | 22 | 14.5 | 10.0 | 16.0 | 30.5 | 50.5 | 7 |
| C3 (*f_w_* dyn, *θ* dyn) | 32.0 | 15.3 | 28 | 18.0 | 14.0 | 21.0 | 39.0 | 64.0 | 9 |

**Table A2: Descriptive statistics for asynchronous call counts per run.** Summary statistics are computed across

*N* = 200 sampled simulation runs for each condition.

| Condition | Mean | SD | Median | IQR | P05 | P25 | P75 P95 | MAD |
| --- | --- | --- | --- | --- | --- | --- | --- | --- |
| C1 (*f_w_* fixed, *θ* fixed) C2 (*f_w_* dyn, *θ* fixed) C3 (*f_w_* dyn, *θ* dyn) | 46.9  32.6  26.5 | 21.2  15.2  11.6 | 42  30  25 | 25.0  20.0  15.0 | 24.0  14.5  12.5 | 31.5  21.0  18.0 | 56.5 100.5  41.0 67.5  33.0 50.0 | 12  10  7 |

**Table A3: Descriptive statistics for asynchronous call fraction per run.** Summary statistics are computed across *N* = 200 sampled simulation runs for each condition.

| Condition | Mean | SD | Median | IQR | P05 | P25 | P75 | P95 | MAD |
| --- | --- | --- | --- | --- | --- | --- | --- | --- | --- |
| C1 (*f_w_* fixed, *θ* fixed) | 0.827 | 0.023 | 0.827 | 0.027 | 0.790 | 0.812 | 0.838 | 0.866 | 0.013 |
| C2 (*f_w_* dyn, *θ* fixed) | 0.569 | 0.065 | 0.571 | 0.097 | 0.467 | 0.520 | 0.617 | 0.672 | 0.047 |
| C3 (*f_w_* dyn, *θ* dyn) | 0.457 | 0.059 | 0.460 | 0.083 | 0.356 | 0.417 | 0.500 | 0.547 | 0.041 |

**Table A4: Descriptive statistics for calls per wingbeat.** Summary statistics are computed across *N* = 200 sampled simulation runs for each condition.

| Condition | Mean | SD | Median | IQR | P05 | P25 | P75 | P95 | MAD |
| --- | --- | --- | --- | --- | --- | --- | --- | --- | --- |
| C1 (*f_w_* fixed, *θ* fixed) C2 (*f_w_* dyn, *θ* fixed) C3 (*f_w_* dyn, *θ* dyn) | 3.028  2.024  1.587 | 1.178  0.305  0.166 | 2.710  1.967  1.562 | 1.418  0.444  0.213 | 1.603  1.622  1.344 | 2.214  1.783  1.475 | 3.633  2.227  1.688 | 5.172  2.556  1.898 | 0.662  0.214  0.102 |

**Table A5: Pairwise effect sizes between conditions.** For each metric, the table reports the difference in medians (CondA*−*CondB) with bootstrap 95% confidence intervals, and Cliff’s *δ* with 95% confidence intervals.

| Metric | Comparison | ∆Median | CI*low* | CI*high* | *δ* | CI*low* | CI*high* |
| --- | --- | --- | --- | --- | --- | --- | --- |
| Synchronous calls (*n*_sync_) | C1–C2 | 13.0 | 11.0 | 15.0 | 0.800 | 0.740 | 0.854 |
| Synchronous calls (*n*_sync_) | C1–C3 | 19.0 | 16.0 | 22.0 | 0.908 | 0.872 | 0.941 |
| Synchronous calls (*n*_sync_) | C2–C3 | 6.0 | 3.0 | 9.5 | 0.302 | 0.198 | 0.410 |
| Asynchronous calls (*n*_async_) | C1–C2 | -12.0 | -16.0 | -8.0 | -0.450 | -0.547 | -0.348 |
| Asynchronous calls (*n*_async_) | C1–C3 | -17.0 | -20.0 | -14.0 | -0.661 | -0.733 | -0.581 |
| Asynchronous calls (*n*_async_) | C2–C3 | -5.0 | -9.0 | -1.0 | -0.240 | -0.345 | -0.126 |
| Async fraction (*f*_async_) | C1–C2 | -0.255 | -0.268 | -0.243 | -1.000 | -1.000 | -1.000 |
| Async fraction (*f*_async_) | C1–C3 | -0.367 | -0.382 | -0.355 | -1.000 | -1.000 | -1.000 |
| Async fraction (*f*_async_) | C2–C3 | -0.111 | -0.131 | -0.094 | -0.786 | -0.842 | -0.722 |
| Calls per wingbeat (*c*_pw_) | C1–C2 | -0.743 | -0.944 | -0.620 | -0.625 | -0.713 | -0.534 |
| Calls per wingbeat (*c*_pw_) | C1–C3 | -1.148 | -1.347 | -1.042 | -0.886 | -0.935 | -0.829 |
| Calls per wingbeat (*c*_pw_) | C2–C3 | -0.404 | -0.477 | -0.340 | -0.829 | -0.876 | -0.774 |

**Table A6: Circular summaries of asynchronous call phases.** Asynchronous call phases are pooled within each condition (total *N*_async_ _phases_ shown). Reported are the mean angle (radians), mean phase as a fraction of the wingbeat cycle, mean resultant length *R*, and circular standard deviation.

Condition *N*_async_ Mean angle (rad) Mean phase (frac.) *R* Circ. SD (rad)

| C1 (*f_w_* fixed, *θ* fixed) | 9377 | 0.188 | 0.0299 | 0.937 | 0.362 |
| --- | --- | --- | --- | --- | --- |
| C2 (*f_w_* dyn, *θ* fixed) | 6516 | 0.130 | 0.0207 | 0.968 | 0.256 |
| C3 (*f_w_* dyn, *θ* dyn) | 5292 | 0.108 | 0.0172 | 0.966 | 0.265 |
